# Clinical Reports from Private Practice

**Published:** 1873-01

**Authors:** 


*Clinical Reports from Private Practice.*

We are pleased to publish an announcement, by Jos. Van Holt Nash, Publisher, Petersburg, Virginia, of "Clinical Reports from Private Practice," by John Herbert Claiborne, M.A., M.D.

From the well-known scientific ability of the author, the profession may expect a work of rare merit. We hail, therefore, with real pleasure the announcement of his work, and predict for it a large circulation. For particulars, the reader is referred to the advertisement of the Publisher, which will be found in our advertising department.

